# Integrated biological–behavioural surveillance in pandemic-threat warning systems

**DOI:** 10.2471/BLT.16.175984

**Published:** 2016-11-03

**Authors:** Maureen Miller, Emily Hagan

**Affiliations:** a Mailman School of Public Health, Columbia University, 722 West 168th Street, New York, NY 10032, United States of America (USA).; b EcoHealth Alliance, New York, USA.

## Abstract

Economically and politically disruptive disease outbreaks are a hallmark of the 21st century. Although pandemics are driven by human behaviours, current surveillance systems for identifying pandemic threats are largely reliant on the monitoring of disease outcomes in clinical settings. Standardized integrated biological–behavioural surveillance could, and should, be used in community settings to complement such clinical monitoring. The usefulness of such an approach has already been demonstrated in studies on human immunodeficiency virus, where integrated surveillance contributed to a biologically based and quantifiable understanding of the behavioural risk factors associated with the transmission dynamics of the virus. When designed according to Strengthening the Reporting of Observational Studies in Epidemiology criteria, integrated surveillance requires that both behavioural risk factors – i.e. exposure variables – and disease-indicator outcome variables be measured in behavioural surveys. In the field of pandemic threats, biological outcome data could address the weaknesses of self-reported data collected in behavioural surveys. Data from serosurveys of viruses with pandemic potential, collected under non-outbreak conditions, indicate that serosurveillance could be used to predict future outbreaks. When conducted together, behavioural surveys and serosurveys could warn of future pandemics, potentially before the disease appears in clinical settings. Traditional disease-outcome surveillance must be frequent and ongoing to remain useful but behavioural surveillance remains informative even if conducted much less often, since behaviour change occurs slowly over time. Only through knowledge of specific behavioural risk factors can interventions and policies that can prevent the next pandemic be developed.

## Résumé

Les épidémies de maladies venant perturber la vie économique et politique sont une caractéristique du XXIe siècle.****Bien que les pandémies soient véhiculées par les comportements humains, les systèmes de surveillance qui permettent actuellement de détecter les menaces pandémiques s'appuient essentiellement sur le suivi des maladies en milieu clinique.****Une surveillance biologico-comportementale intégrée et standardisée pourrait et devrait être appliquée dans les communautés, en complément de ce suivi clinique.****L'utilité de ce type d'approche a déjà été démontrée dans des études sur le virus de l'immunodéficience humaine, où la surveillance intégrée a permis de comprendre et de quantifier, sur la base d'éléments biologiques, les facteurs de risque comportementaux associés à la dynamique de transmission du virus. En application des critères STROBE (Strengthening the Reporting of Observational Studies in Epidemiology), la surveillance intégrée exige que soient mesurés à la fois les facteurs de risque comportementaux - à savoir les variables d'exposition - et les variables d'indicateurs de la maladie lors d'enquêtes comportementales. Dans le domaine des menaces pandémiques, les données biologiques pourraient compenser les faiblesses des données recueillies lors des enquêtes comportementales.****Les données issues d'enquêtes sérologiques sur des virus susceptibles de causer des pandémies, recueillies hors épidémie, indiquent que la surveillance sérologique pourrait être utilisée pour prédire les futures épidémies. Réalisées conjointement, les enquêtes comportementales et les enquêtes sérologiques pourraient avertir des futures pandémies, potentiellement avant que les maladies n'apparaissent en milieu clinique. La surveillance traditionnelle des maladies doit être fréquente et continue pour rester utile, mais la surveillance comportementale reste informative même si elle est effectuée moins souvent, car les changements de comportement s'effectuent beaucoup plus lentement. Les interventions et les politiques pouvant empêcher une prochaine pandémie ne peuvent être élaborées qu'en connaissant spécifiquement les facteurs de risque comportementaux.

## Resumen

Los brotes de enfermedades perjudiciales para la economía y la política son una característica del siglo XXI. A pesar de que las pandemias se ven impulsadas por el comportamiento humano, los sistemas de vigilancia actuales para identificar amenazas de pandemia dependen enormemente del seguimiento de los resultados de las enfermedades en entornos clínicos. La vigilancia integrada y normalizada de datos biológicos y del comportamiento podría y debería utilizarse en comunidades como complemento de dicho seguimiento clínico. Su utilidad ya se ha demostrado en varios estudios sobre el virus de la inmunodeficiencia humana, en los que la vigilancia integrada contribuyó a la comprensión cuantificable y biológica de los factores de riesgo del comportamiento asociados con la dinámica de la transmisión del virus. Al estar diseñada según los criterios del Fortalecimiento de la Notificación de los Estudios Observacionales en Epidemiología, la vigilancia integrada requiere tanto los factores de riesgo del comportamiento (es decir, las variables de exposición) como las variables de resultados del indicador de enfermedades se midan en encuestas sobre el comportamiento. En el campo de las amenazas de pandemia, los datos de los resultados biológicos podrían abordar la debilidad de los datos autodeclarados recopilados en las encuestas sobre el comportamiento. La información de las encuestas serológicas sobre virus con potencial pandémico, recopilada en condiciones en las que no se había reportado un brote, indica que podría utilizarse la vigilancia serológica para predecir futuros brotes. Al realizarse juntas, las encuestas serológicas y sobre el comportamiento podrían advertir sobre futuras pandemias, probablemente antes de que la enfermedad aparezca en entornos clínicos. La vigilancia tradicional de los resultados de las enfermedades debe ser constante y frecuente para que sea útil, aunque la vigilancia sobre el comportamiento sigue siendo meramente informativa, incluso si se realiza con menos asiduidad, dado que los cambios de comportamiento se producen lentamente con el paso del tiempo. Únicamente a través del conocimiento de los factores de riesgo del comportamiento específicos se pueden desarrollar las intervenciones y las políticas capaces de evitar el desarrollo de la próxima pandemia.

## ملخص

إن حالات تفشي الأمراض الخطيرة وتأثيرها على الوضع الاقتصادي والسياسي هي السمة المميزة للقرن الحادي والعشرين. بالرغم من أن الأوبئة ناتجة عن السلوكيات البشرية، إلا أن أنظمة المراقبة الحالية لتحديد أخطار الأوبئة تعتمد بشكل كبير على رصد حصائل المرض في المواقع العلاجية. وهناك إمكانية – بل واستحسان – لاستخدام وسائل المراقبة البيولوجية والسلوكية القياسية في البيئات المجتمعية لاستكمال حالات رصد التجارب العلاجية من هذا النوع. لقد تم بالفعل استعراض مدى جدوى اتباع مثل هذا النهج في الدراسات التي تم إعدادها بخصوص فيروس عوز المناعة البشري، حيث ساهمت المراقبة المتكاملة في استيعاب عوامل الخطورة السلوكية المرتبطة بديناميكيات انتقال الفيروس بطريقة قائمة على الناحية البيولوجية والقابلية للقياس الكمي. تتطلب المراقبة المتكاملة أن يتم قياس كل من عوامل الخطورة السلوكية – على سبيل المثال متغيرات التعرض للمرض ومتغيرات حصيلة مؤشرات المرض في المسوح السلوكية، وذلك عندما يتم إعدادها وفقًا لمعايير "تقوية الإبلاغ عن الدراسات الرقابية في الوبائيات". في مجال مخاطر الأوبئة، يمكن أن تعالج بيانات الحصائل البيولوجية مواطن الضعف الخاصة بالبيانات المُبلّغ عنها ذاتيًا والتي تم تجميعها في المسوح السلوكية. كما أن البيانات الناتجة عن المسوح المصلية للفيروسات القادرة على إحداث وباء، والتي تم جمعها في ظل ظروف ينعدم فيها تفشي الأمراض تشير إلى أنه بالإمكان استخدام المراقبة المصلية للتنبؤ بحالات تفشي الأمراض في المستقبل. وعند إجراء المسوح السلوكية والمسوح المصلية معًا، فبإمكانها أن تكون أدوات للتحذير من انتشار الأوبئة في المستقبل، وربما قبل ظهور المرض في المواقع العلاجية. يجب أن تكون عملية مراقبة حصيلة المرض التقليدية متكررة ومستمرة لتظل ذات فائدة ولكن المراقبة السلوكية لاتزال غنية بالمعلومات المفيدة حتى لو أجريت في أوقات أقل بكثير، وذلك لأن التغيير السلوكي يحدث ببطء على مدار الزمن. ولا ينبغي إعداد التدخلات والسياسات التي يمكنها منع حدوث الوباء القادم إلا من خلال معرفة عوامل الخطر السلوكية المحددة.

## 摘要

经济和政治性破坏导致疾病的爆发是 21 世纪的一个标志。尽管流行病是由于人类活动造成的，但目前用于确定流行病威胁的监督系统很大程度上依赖于临床环境中疾病效果的监测情况。标准化综合生物学行为监督可以并且应该应用于社区环境中，对此类临床监督进行补充。这类方法的有效性已经在与人类免疫缺陷病毒有关的研究中阐述过。该研究指出，综合监督有助于从基于生物学和定量的角度理解与病毒传播动态有关的行为风险因素。根据“加强流行病观察研究报告”标准进行设计之后，综合监督需要在行为调查中同时衡量行为风险因素（即，接触变量）和疾病指标结果变量。在流行病威胁方面，生物学结果数据可解决行为调查中采集的自我报告式数据的缺陷问题。在尚未爆发的状态下采集的潜在流行病毒的血清调查数据显示，血清监督可用于预测未来流行病的爆发。在同时开展的前提下，行为调查和血清调查可能会在临床发现疾病之前对未来流行病提出预警。传统疾病结果监督必须定期且持续进行才能保持有效性，但行为监督即使是在开展频率较低的情况下依然能够提供有益信息，因为随着时间推移，行为变化的进程很缓慢。只有掌握具体行为风险因素才能制定出可预防下一场流行病爆发的干预和政策。

## Резюме

Отличительной чертой 21-го века являются вспышки заболеваний, оказывающие разрушительный эффект на экономику и политику. Хотя причины пандемий кроются в поведении людей, современные системы наблюдения, предназначенные для выявления угроз пандемии, в значительной мере полагаются на мониторинг исходов заболевания в клинических условиях. Стандартизованное, комплексное, биологически поведенческое наблюдение можно и следует применять в условиях общин как дополнение к подобному клиническому мониторингу. Целесообразность такого подхода уже была продемонстрирована в исследованиях вируса иммунодефицита человека, в рамках которых комплексное наблюдение способствовало биологически обоснованному и поддающемуся количественной оценке пониманию факторов поведенческого риска, связанных с динамикой передачи вируса. Для комплексного наблюдения, разработанного в соответствии с критериями устранения недостатков предоставления информации в наблюдательных исследованиях по эпидемиологии, необходимо измерение факторов поведенческого риска, т. е. переменных подверженности воздействию, и выходных переменных индикаторов заболеваемости в рамках исследований поведения. В сфере угроз пандемии с помощью информации о биологических исходах можно было бы устранить недостатки предоставленных респондентами данных, полученных в рамках поведенческих исследований. Судя по данным серологических исследований вирусов, имеющих пандемический потенциал, которые были получены в условиях отсутствия вспышки, эпидемиологический надзор может быть использован для прогнозирования будущих вспышек. Проведение поведенческих и серологических исследований вместе позволило бы спрогнозировать будущие пандемии теоретически до того, как заболевание возникнет в клинических условиях. Польза традиционного наблюдения исходов заболеваний зависит от частоты и непрерывности его проведения, в то время как поведенческое наблюдение позволяет получить ценную информацию, даже если осуществляется гораздо реже, поскольку поведение со временем изменяется медленно. Только зная конкретные факторы поведенческого риска, можно разработать мероприятия и политики, способные предотвращать будущие пандемии.

## Introduction

No other modern epidemic or pandemic mobilized the global health community to action like the 2013–2016 Ebola virus disease outbreak in western Africa. Following the outbreak, calls for pandemic-threat warning systems came from both traditional public health policy-makers^1^^,^^2^ and national governments.^3^ As currently conceptualized, the first step in the identification of a pandemic threat requires an outbreak of sufficient size to come to the attention of medical personnel who are sufficiently influential and persistent to ensure action.^4^ Once an outbreak is verified, well-established protocols for disease investigation and control can be swiftly put in place – although it may be many months before the main risk factors and most effective control measures are identified. The Ebola outbreak in western Africa probably began in December 2013^5^ but it took another year before traditional burial practices were found to be a leading cause of the rapid spread of the causative virus.^6^

## Monitoring emerging infectious diseases

Although human behaviours often increase the risk of acquiring an infectious disease, the systematic investigation of human risk behaviours is seldom included in disease surveillance strategies.^7^ However, behavioural surveillance to improve the understanding of human immunodeficiency virus (HIV) and acquired immunodeficiency syndrome has been ongoing for decades. Behavioural assessment was key to the identification of injecting drug users as a high-risk group for HIV infection in the early 1980s.^8^ It was also crucial in documenting the risks posed by HIV to women.^9^ Subsequently, an innovative, practical method, which combines biological outcome data with behavioural risk factor data – i.e. exposure variables – was developed to document HIV transmission dynamics. Such integrated biological–behavioural surveillance has since become well established and standardized and been frequently implemented globally.^10^^,^^11^ It has contributed extensively to a biologically-based and quantifiable understanding of the behavioural risk factors associated with the acquisition and transmission of HIV^12^ and the early identification of subgroups of the population that may be more vulnerable to HIV infection.^9^ More recently, data from integrated surveillance have been used to evaluate the impact of evidence-based interventions to prevent HIV infection and to monitor treatment uptake.^13^ Similar surveillance could help identify behavioural risk factors and high-risk subgroups for zoonotic infections such as Ebola – potentially before diseases of pandemic potential are identified in clinical settings or major outbreaks occur in communities.

Approximately half of the emerging pandemic threats are zoonotic in origin.^14^^,^^15^ At the time of writing, the most lethal and costly pandemics of the 21st century, i.e. avian influenza, Ebola, Middle East respiratory syndrome (MERS) and severe acute respiratory syndrome (SARS), have all been caused by zoonotic viruses.^16^^–^^19^ Little is known about either the risk factors that lead to the initial spillover of a zoonotic disease into human populations or the dynamics of any subsequent human-to-human transmission.^20^ Much more is known about (i) the locations of so-called hotspots where, many scientists believe, new pandemics of zoonotic disease are likely to emerge;^14^ (ii) the kinds of ecological and environmental activities that have been associated with spillover and outbreaks of zoonotic disease in the past;^21^ and (iii) the distinct spatial groupings of specific infectious diseases on a global scale, and the associated ecological and virological barriers to the dispersal and establishment of those diseases.^22^ In the development of pandemic-threat warning systems, integrated biological–behavioural surveillance can be tightly focused on specific viral families in the high-risk population subgroups that live in identified hotspots and are environmentally or occupationally exposed to animals.

The remainder of this article presents an overview of issues relevant to the design of rigorous behavioural surveys to assess the spillover of emerging zoonotic disease and the associated transmission risk factors, which is the first step in designing effective integrated surveillance. We identified community-based serological surveys of viruses with pandemic potential as a possible source of useful biological outcome data. We summarize the results of such serosurveys, conducted in non-outbreak settings in Africa, and evaluate their usefulness – especially when used in combination with behavioural surveillance – in the prediction of future outbreaks.

## Designing behavioural risk surveys

When designed according to Strengthening the Reporting of Observational Studies in Epidemiology criteria,^23^ integrated surveillance requires that both disease-indicator outcome variables and behavioural risk factors be measured in behavioural surveys. Behavioural risk factors, i.e. exposure variables, simply represent the population prevalence of behaviours that may or may not increase the risk of disease. Without the outcome variables, the exposure variables are of little use in elucidating the mechanisms of the spillover of zoonotic disease to humans or of subsequent human-to-human transmission. Effective surveillance requires questions that assess a range of animal exposures, document experiences of unusual illness and measure contextual factors that can lead to an increase or decrease in the probabilities of behavioural risk factors and disease.

In studies on zoonoses, the assessment of behavioural risk factors is complicated because different zoonotic diseases may be associated with different kinds of animal exposure. The spillover of zoonotic viruses from wildlife, the source of most emerging zoonoses,^15^ has been difficult to document. Behavioural risk may be either direct or indirect. Direct contact with primate blood or bodily fluids has been associated with several zoonotic viruses found in humans, such as human T-lymphocyte virus,^24^ simian foamy virus^25^^,^^26^ and, possibly, Ebola.^5^ Indirect contact was responsible for the transmission of Nipah virus to humans, which was mediated through date-palm sap contaminated with the urine of infected bats,^27^ and hantavirus transmission is most frequently associated with inhalation of aerosolized virus from the excreta of infected rodents.^28^ Both general exposure to animals, e.g. when buying live animals at market, and more intimate exposure, e.g. during the slaughter of animals or as the result of animal bites, must therefore be assessed.

Syndromic surveillance is widely used to monitor illnesses of unknown etiology in clinical settings and can provide a useful referent in the identification of the outcome variables to be measured in behavioural surveys. Several zoonotic diseases, such as avian influenza,^17^ MERS^16^ and SARS,^29^ were identified via syndromic surveillance networks, from localized increases in the incidence of influenza-like illness or severe acute respiratory infection. By using standardized definitions, it should be easy to develop questions or symptom checklists for syndromic surveillance based on self-reported data. Such definitions already exist for influenza-like illness,^30^ severe acute respiratory infection^30^ and other syndromes consistent with zoonotic infection, such as encephalitis of unknown origin,^31^ fever of unknown origin^32^ and haemorrhagic fever of unknown origin.^33^

The risks posed to humans by exposure to animals may be modified by various biological, ecological, economic, political and sociocultural factors.^34^ For example, poverty can place individuals and communities at the forefront of zoonotic disease risk through several mechanisms. Exposure to dense or diverse rodent populations in urban or rural environments^35^^,^^36^ and the displacement of wildlife populations as land is cleared for crops are some mechanisms.^36^ Understanding the context within which spillover to humans can occur is an important component in the prevention of zoonotic outbreaks. The same behavioural risk factors may be risky in one context but not in another. For instance, the sharing of a water source with animals displaced by a change in land use may only have an adverse effect on human health if there is faecal–oral transmission of the zoonotic pathogen to humans. If such transmission requires contact with the animal blood, then the sharing of the water source should not increase the risks of either spillover or transmission. Once human-to-human transmission of a zoonotic pathogen occurs, additional risks come into play, often as a consequence of the human infection, and these should also be measured pre-emptively. Burial practices and health-care-seeking practices were associated with explosive increases in, respectively, the incidence of human infection with Ebola virus in western Africa^6^ and MERS in the Republic of Korea.^37^

Even in the absence of detailed biological outcome data, behavioural surveillance may be used to assess relationships between behavioural risk factors and self-reported experiences of unusual illness that are consistent with the symptoms of zoonotic disease. This could be done both rapidly and at a scale that facilitates epidemiologically relevant analyses. Although not as conclusive as biological data, self-reported data could provide substantially more information than is currently available.

## Non-outbreak serosurveys

In the field of pandemic threats, biological outcome data could address the weaknesses of any self-reported data collected in behavioural surveys. The ideal source of biological outcome data, i.e. data that provide the strongest evidence of zoonotic disease spillover, would be community-based screening for acute infection with zoonotic viruses. However, as such infection is rare under non-outbreak conditions, many thousands of individuals would usually have to be screened for a meaningful analysis of the behavioural risk factors. Serological assays, in which previous exposure to a virus is demonstrated by a positive result, can provide alternative biological outcome data. Since many more individuals may have been exposed to a virus than are currently ill with the virus, serology can provide the larger number of individuals, with known viral exposure, required for powerful analyses of behavioural risk factors. We therefore investigated the results of serosurveys for their potential usefulness in the prediction of future outbreaks. We focused on studies conducted in communities under non-outbreak settings in Africa. We collated results presented in peer-reviewed publications – in English, French or Spanish – that we identified via Google Scholar and Web of Science searches that ended on 31 December 2015 (available from the corresponding author). We used “Africa”, “antibody”, “serology”, “serosurvey”, “zoonoses” and “zoonotic disease” as search terms. We identified additional relevant results through the citations in the articles identified in the searches.

Serosurveys of zoonotic viruses have been conducted since the discovery of the Ebola virus in the 1970s, mostly during or shortly after an outbreak of zoonotic disease. Our searches revealed 38 serosurveys of zoonotic viruses in Africa that had been conducted during non-outbreak conditions.^25^^,^^26^^,^^38^^–^^49^ To identify any associations between population subgroup risk and seroprevalence, we divided the subjects of the serosurveys into three risk categories, based on the limited data from previous research on zoonotic disease spillover. For example, hunters have been consistently found to be a high-risk population subgroup,^25^^,^^26^^,^^42^^,^^45^^,^^48^ followed by rural populations, who have been categorized as medium-risk because of their close and regular proximity to wildlife.^38^^,^^39^^,^^41^^,^^43^^,^^44^^,^^46^^,^^49^ Randomized or representative samples of general populations^40^^,^^47^ have been considered to be low-risk.

Serological assays for several different zoonotic pathogens were conducted as part of serosurveillance in each of the 14 studies included in our analysis. Use of these assays led to the recording of a total of 38 unique zoonotic pathogen seroprevalences that ranged from 0% to 24%. Of these 38 seroprevalences, nine were recorded for high-risk population subgroups, 19 for medium-risk and 10 for low-risk. Evidence of previous exposure to a zoonotic pathogen, i.e. a seroprevalence of more than zero, was detected in eight (89%) of the high-risk population subgroups, 16 (84%) of the medium-risk and seven (70%) of the low-risk (available from the corresponding author). High seroprevalences, i.e. seroprevalences of at least 1%, represented the results for all eight of the high-risk subgroups with evidence of previous exposure to a zoonotic pathogen, 12 (75%) of the medium-risk subgroups with such evidence and three (48%) of the low-risk subgroups with such evidence (available from the corresponding author). Exposure to wildlife therefore appeared to be associated both with any evidence of viral exposure and with high seroprevalence.

Since the first known outbreak in 1976, the United States Centers for Disease Control and Prevention have recorded 35 documented Ebola outbreaks in Africa.^50^ More than 5% of the subjects included in serosurveys in Gabon in 1981^43^ and Liberia in 1982^46^ showed evidence of exposure to Ebola virus, that is decades before an Ebola outbreak was first reported in either of these countries. Between 1.9% and 12.4% of the subjects included in Ebola serosurveys in three countries that have never reported an Ebola outbreak, the Central African Republic,^40^^,^^44^^,^^48^ Madagascar^47^ and Zimbabwe,^39^ were also found to be seropositive.

Although serological assays exist for the coronaviruses that cause MERS and SARS, Hantaan viruses and paramyxoviruses, most serosurveys of zoonotic viruses in Africa have focused on haemorrhagic fevers. Most of the serosurveys we reviewed had also been done before the widespread availability and use of viral detection tests. Recently, population-based serosurveys have been increasingly adopted, in recognition of their utility in preparing health authorities for potential outbreaks or epidemics.^26^^,^^38^^,^^49^ Current serological assay methods tend to be labour-intensive and to suffer from cross-reactivity that prevents distinction between several antigenically related viruses. However, the last few years have witnessed major advances in the development of economically feasible methods for comprehensive serological profiling^51^^,^^52^ and these should facilitate the investigation of zoonotic spillover into human populations.

## Prediction and risk mitigation

We review the potential contributions that integrated biological–behavioural surveillance could make to pandemic-threat prediction, prevention and risk mitigation. If we are to mitigate the risk of a zoonotic disease outbreak, we need a better understanding of the mechanisms behind the spillover of zoonotic disease into human populations. By making such mechanisms the focus of integrated surveillance, we should be able to: (i) monitor the presence and prevalence of behavioural risk factors and the seroprevalence of specific zoonotic pathogens within particular population subgroups; (ii) deploy targeted control and mitigation strategies rapidly; and (iii) evaluate the efficacy of prevention policies and interventions.

Although traditional disease surveillance must be frequent and ongoing to remain useful, behavioural surveillance remains informative even if conducted much less often, since behaviour change occurs slowly over time. To be effective as a prevention tool, integrated biological–behavioural surveillance should be implemented as a baseline measure – to identify behavioural risk factors, determine the prevalence of those risk factors, especially in any population subgroups that are considered at higher risk of zoonotic spillover, and establish seroprevalence. Should an outbreak occur, a database that documents local behaviours and practices, as well as the context within which such behaviours occur, can contribute to the development of appropriate and feasible strategies for disease control and mitigation. Finally, data from integrated surveillance will be invaluable in both informing realistic and effective interventions and policies for the prevention of zoonotic spillover and transmission, and evaluating the impact of such interventions and policies efficiently.

## Preventing the next pandemic

Relative to the economic, social and political costs of epidemics, prevention will always be less expensive^3^^,^^19^ if the targets of prevention activities are well understood and acted upon. The fact that success can feel more like the status quo is a challenge unique to prevention. The political commitment for prevention activities will often be less than that for a reactive response elicited by the emergence of a terrifying new infectious disease. However, political support may be improved if surveillance is made to be, and appear, more cost–effective, by focusing on specific diseases^22^ in population subgroups who live in ecologically fragile hotspots.^14^^,^^21^

There is substantial overlap between areas considered to be hotspots for zoonotic disease spillover and those considered hotspots for HIV, perhaps the best known zoonotic virus.^14^^,^^53^ This overlap opens a real possibility of merging attempts to detect zoonotic disease spillover with pre-existing population-based systems that have been used to investigate the HIV epidemic for several decades. For example, the Demographic and Health Surveys Program is implemented globally in settings without high-quality civil registration and has extensive experience in collecting integrated biological–behavioural surveillance data in community settings.^54^

Integrated surveillance will never be a viable alternative to traditional clinical disease surveillance for assessing active viral infections. Rather, it can serve to complement virus detection efforts, by potentially identifying pandemic threats before the need for large-scale clinical intervention. As current behaviours may not reflect the behaviours that originally exposed the individuals who are found seropositive to the virus of interest, both current and lifetime behaviours need to be investigated. This is the strategy that has proved successful in identifying subtle exposure risks in the field of HIV, such as backloading of syringes with drug solution by injecting drug users.^55^ In identifying specific behavioural risk factors, integrated biological–behavioural surveillance will be most effective when the reported syndromic symptoms are recent, e.g. occurring in the previous 12 months, and their probable association with a zoonotic virus can be confirmed by a positive serological test result.

## Conclusion

Current pandemic-threat warning systems rely almost exclusively on disease surveillance in clinical settings. Standardized biological–behavioural surveillance, in which both disease outcome data – self-reported and biological – and behavioural risk factors are measured, would complement traditional surveillance and greatly advance the understanding of behaviours and practices that could be targeted for risk mitigation and, ultimately, for prevention. The implementation of integrated biological–behavioural surveillance need not be frequent to be informative and useful in preventing the spillover of zoonotic agents with pandemic potential.
